# Rethinking Guideline Delivery in Nephrology: From Access to Action

**DOI:** 10.1016/j.ekir.2026.107089

**Published:** 2026-09-21

**Authors:** Rob C.M. van Kruijsdijk, Tobias T. Pieters, Joel M. Topf, James O. Burton

**Affiliations:** 1 Department of Nephrology, Radboud University Medical Center, Nijmegen, The Netherlands; 2 Department of Medicine, Oakland University William Beaumont School of Medicine, Rochester, Michigan, USA; 3 John Walls Renal Unit, Leicester General Hospital, Leicester, UK; 4 Division of Cardiovascular Sciences, National Institute for Health and Care Research (NIHR) Leicester Cardiovascular Biomedical Research Unit, University of Leicester, Leicester, UK

Clinical practice guidelines play a central role in improving the quality, consistency, and evidence-based nature of nephrology care. However, despite the proliferation of high-quality guidelines, their translation into daily clinical practice remains suboptimal.^1^^,^^2^ These gaps between evidence and implementation contribute to undesirable variation in care and outcomes among patients with kidney disease.

Clinicians often face limited time, information overload, and increasingly complex workflows that may hinder their ability to access and apply guideline recommendations at the point of care. Although static formats such as online portable document format documents ensure broad availability, they lack efficient navigation, contextual adaptation, and real-time integration into electronic health records (EHRs) or other clinical systems. Furthermore, as guideline organizations employ a limited range of standard dissemination strategies chosen primarily for feasibility, guideline impact could be enhanced through more structured implementation planning and systematic evaluation.^3^ A range of digital approaches has been proposed to help overcome these limitations, offering opportunities to make guidelines more accessible, usable, and actionable within contemporary clinical workflows. We use the term “digital approaches” broadly, spanning a spectrum from static portable document formats and webpages to modular guideline platforms, EHR-integrated decision support, mobile applications, and artificial intelligence-assisted tools, as well as digital channels for dissemination and education. Drawing on a literature search and on reach and usage data from nephrology guideline organizations and digital education platforms (Supplementary Methods), we evaluate the accessibility, usability, and uptake of these approaches, identify key barriers and facilitators to implementation, and outline evidence-informed directions for future digital guideline delivery in nephrology, ultimately to improve patient outcomes. A more detailed discussion of the relevant studies is provided in Supplementary Text.

### Web-Based Guideline Platforms

Clinical practice guidelines are still primarily disseminated through static formats, which ensure open access but offer limited functionality for navigation, updating, or detailed usage monitoring. Within initiatives such as Developing and Evaluating Communication strategies to support Informed Decisions and practice based on Evidence and Making GRADE the Irresistible Choice, guideline content has been developed into modular and interactive web-based formats, using a multilayered electronic presentation in which clinicians can progressively expand recommendations to view their rationale and concise evidence summaries.S1^,^S2 In a cluster-randomized trial comparing a traditional print and portable document format with an interactive MAGIC application-based format, no differences were observed in knowledge gain, but users of the interactive format reported better ease of use, faster learning, and greater improvements in decision-making.^4^ In parallel, the National Institute for Health and Care Excellence has described a transition to natively digital, computable, and modular guideline content.^5^ Within nephrology, Kidney Disease: Improving Global Outcomes (KDIGO) has adopted a modular update approach for selected guidelines, revising the 2021 Glomerular Diseases Guideline chapter by chapter in order to respond more rapidly to new trial evidence and drug approvals.^6^ Such modular web-based architectures enable continuous updating and linkage with clinical data systems, thereby providing the technical foundation for truly living guideline models.S3

For KDIGO guidelines, page views and portable document format downloads via the organization’s website substantially exceeded access via the journal website (Table 1)S4–S19, suggesting that the organizational website functions as the primary point of entry for guideline content for most users. User-reported preferences from a global KDIGO guideline survey (*n* = 1018) similarly indicate that concise, structured formats are most valued, with executive summaries and top 10 takeaways rated highest for practical utility in clinical practice (KDIGO, Global Survey on Utilization and Implementation of KDIGO Guidelines, unpublished data, 2025).

**Table 1:** Reach and usage metrics across digital nephrology guideline dissemination and education platforms

Guideline	Publication date	Organization website					Journal website	NephJC Online Journal Club		Freely Filtered podcast		
		Page views	PDF downloads	Executive Summary	Data supplement	Guideline suite: key takeaways	Full Guideline	Date	Page views	Date	Total plays^a^	Engaged audience^b^
UKKA 2025 Blood Pressure in DialysisS4	26 Sep 2025	-	-	-	-	-	1860 (365)	-	-	-	-	-
KDIGO 2025 IgAN/IgAVS5	18 Sep 2025	181,194 (33,838)	29,581 (5524)	17,549 (3277)	2496 (466)	26,620 (4971)	-	28 Oct 2025	5592 (1384)	-	-	-
UKKA 2025 Vascular Access for HDS6	14 Aug 2025	-	-	-	-	-	5750 (884)	-	-	-	-	-
UKKA 2025 Anaemia of CKDS7	16 Apr 2025	-	-	-	-	-	7965 (762)	-	-	-	-	-
KDIGO 2025 ADPKDS8	21 Jan 2025	717,475 (54,193)	31,524 (2381)	14,961 (1130)	2989 (226)	5331 (403)	-	25 Mar 2025	6715 (601)	12 May 2025	12,456 (1298)	2004 (209)
ABCD-UKKA 2024 Lipids in Diabetic KDS9	6 Jul 2024	-	-	-	-	-	12,000 (607)	-	-	-	-	-
KDIGO 2024 CKDS10	13 Mar 2024	1,494,276 (63,439)	329,216 (13,977)	105,097 (4462)	17,620 (748)	143,798 (6105)	-	7 & 21 May 2024^c^	78,899 (3628)	1 Aug 2024	28,314 (1496)	2994 (158)
KDIGO 2024 Lupus NephritisS11	3 Jan 2024	216,782 (8385)	107,665 (4164)	110,597 (4278)	5710 (221)	24,998 (967)	-	6 Feb 2024	6595 (267)	-	-	-
ISPD 2022 PeritonitisS12	10 Mar 2022	-	-	-	-	-	798,727 (16,756)	19 Apr 2022	35,014 (755)	13 May 2022	18,882 (414)	1685 (37)
KDIGO 2021 Glomerular DiseaseS13	20 Sep 2021	503,007 (9440)	207,183 (3888)	53,051 (996)	8395 (158)	62,828 (1179)	-	16 Nov 2021	18,741 (365)	-	-	-
KDIGO 2021 Blood Pressure in CKDS14	23 Feb 2021	859,446 (14,288)	323,039 (5370)	60,381 (1004)	10,772 (179)	32,581 (542)	-	8 Jun 2021	13,061 (230)	25 Jul 2021	25,563 (463)	2057 (37)
KDIGO 2020 Diabetes in CKDS15	30 Sep 2020	421,122 (6484)	152,341 (2346)	53,185 (819)	8480 (131)	32,904 (507)	-	20 Oct 2020	7417 (115)	28 Nov 2020	21,965 (349)	1574 (25)
ISPD PD in AKI 2020 Update (pediatrics)S16	1 Feb 2021	-	-	-	-	-	47,902 (787)	9 Mar 2021	11,053 (185)	-	-	-
AHA 2017 HypertensionS17	13 Nov 2017	-	-	-	-	-	2,485,755 (24,981)	16 Jan 2018	9875 (101)	-	-	-
ESICM, ESE, ERA-EDTA 2014 HyponaetremiaS18^,^S19	1 Mar 2014	-	-	-	-	-	273,761 (1902)	10 Jun 2014	57,384 (408)	-	-	-

ABCD-UKKA, Association of British Clinical Diabetologists–UK Kidney Association; ADPKD, autosomal dominant polycystic kidney disease; AHA, American Heart Association; CKD, chronic kidney disease; EHR, electronic health record; ERA-EDTA, European Renal Association–European Dialysis and Transplant Association; ESICM, European Society of Intensive Care Medicine; ESE, European Society of Endocrinology; HDS, hemodialysis; IgAN, IgA nephropathy; IgAV, IgA vasculitis; ISPD, International Society for Peritoneal Dialysis; KDIGO, Kidney Disease: Improving Global Outcomes; KDS, kidney disease; NephJC, Nephrology Journal Club; PD, peritoneal dialysis; UKKA, UK Kidney Association.

Data are displayed as cumulative totals through February 28, 2026 (views per downloads per month since first availability). Data include page views and downloads combined unless otherwise indicated and reflect content-access events, not unique users; see Supplementary Methods for platform-specific counting methodology.

a Total number of episode plays across Apple and Spotify.

b Total number of listeners meeting platform-defined engagement thresholds (Apple ≥ 20 min/≥ 40% of episode; Spotify ≥ 30 second)

c Part 1 and 2 combined; the first of the 2 dates was used to calculate views per month.

### Interactive Tools and EHR-Integrated Decision Support

Efforts to transform static guideline documents into interactive, user-oriented tools that support clinical reasoning and case-specific recommendations have produced digital checklists and forms, visual encounter aids and web-based clinical decision support systems. These are often built on knowledge-modeling approaches in which guideline logic is translated into computer-readable formats that can be linked to patient data and clinical workflows.S20

Empirical studies across diverse settings demonstrate how interactivity and contextualization can enhance decision quality and adherence. For example, in a randomized trial, an online HIV treatment platform doubled the accuracy of regimen selection compared with static guideline documents.S21 Similar checklist-, encounter-aid-, and model-driven tools have improved compliance across intensive care, obstetrical, primary care, and pediatric settings, although their effects were often context-dependent and limited to process measures rather than hard outcomes.S22–S26

Integrating clinical guidelines within EHRs represents a crucial step toward bringing evidence-based recommendations directly to the point of care. For example, in China, a pragmatic cluster-randomized trial showed that a decision-support system providing real-time, patient-specific hypertension advice increased adherence to recommended therapy by 15.2%.^7^ However, technical integration alone is not sufficient. For example, the Nationwide Utilization of Danish Government Electronic Letter System for Increasing Guideline-Directed Medical Therapy in Chronic Kidney Disease trial, using digital letters to encourage patients with chronic kidney disease and their general practitioners to start recommended therapies, showed no effect on prescribing rates, as the intervention was neither embedded in the EHR nor personalized to clinical encounters.^8^ Similar contrasts were seen across other alert-, order-set-, and feedback-based interventions, with more deeply embedded and better-timed approaches most consistently improving adherence.S27–S35 Strengths and limitations of each approach are summarized in Supplementary Table S1.

### Mobile Applications

Mobile applications offer a promising platform for delivering guideline-derived algorithms, risk scores, and clinical decision support systems during clinical care and could be particularly useful in time-pressured environments or in low-resource contexts.S36–S40 Alongside these clinician-facing tools, patient-oriented self-management apps may improve self-management behaviors, blood-pressure control, and medication adherence in patients with chronic kidney disease, although supporting studies vary in quality and duration.S41–S43 Initial uptake is often high, but long-term engagement declines,S44–S46 and clinicians report that applicationss perceived as outdated, slow, or poorly aligned with their routines quickly fall out of use. Nonetheless, demand exists: in the KDIGO guideline survey, 80.5% indicated that a mobile application with easy access to guidelines would be a value-added resource (KDIGO, unpublished data, 2025). At present, no peer-reviewed articles examine the uptake, implementation, or clinical effect of the major nephrology guideline-based applications.

### Artificial Intelligence

Artificial intelligence holds considerable promise for guideline dissemination, supporting clinicians with tools ranging from simple extraction of relevant recommendations to full decision support and supporting patients through plain-language explanations. However, the pace of artificial intelligence development makes it unlikely that peer-reviewed research can keep up. Despite this, artificial intelligence models are already used widely in clinical practice, with a recent American Medical Association survey indicating that 66% of physicians report using artificial intelligence tools regularly.S47

Several studies compare general-purpose large language models in tasks like staging cancers, recommending treatment plans, or generating patient management advice,S48–S53 with generally moderate-to-high guideline adherence reported across oncology use casesS53–S56 and more heterogeneous results in other clinical domains.S49^,^S51 Retrieval-augmented generation systems that integrate guideline text directly into the model context enable more transparent and verifiable outputs and reduce hallucinations,S57–S59 and large language models can automatically extract key eligibility criteria and recommended actions from guidelines, enabling the generation of structured decision support logic.S60 However, in common hypertension management scenarios, accuracy and safety remained inferior to expert decisions, underscoring the need for human-in-the-loop oversight.S61 Most studies relied on simulated vignettes, and none compared these systems with established rule-based decision support tools. Stronger clinical evidence and standardized evaluation are needed before routine implementation can be recommended.

### Digital Dissemination and Education Tools

Beyond decision-support technologies, a growing ecosystem of webinars, online journal clubs, podcasts, and microlearning formats enable low-threshold engagement with guideline content. Within nephrology, social-media–enabled education initiatives such as Nephrology Journal Club exemplify how online journal clubs can achieve sustained international participation and broad dissemination, extending critical appraisal and discussion well beyond local institutions.^9^^,^S62^,^S63 Podcasts are valued by clinicians for their accessibility, flexibility, and ability to contextualize complex topics.S64 Although microlearning approaches are associated with high learner engagement and short-term gains in knowledge and confidence, the effects on clinician behavior are rarely evaluated.S65 Table 1 illustrates that these formats can achieve substantial reach, and the KDIGO guideline survey indicates substantial demand, with online training modules (53%) and educational webinars (42%) among the most frequently requested additional resources (KDIGO, unpublished data, 2025).

### Barriers and Facilitators

Across these studies, digital tools are most effective when they are delivered at the moment of decision, provide concise and patient-specific recommendations, and link directly to executable actions such as orders or documentation templates.^7^^,^S28^,^S30–S32 Tools that operate outside the clinical workflow or rely on passive reminders rarely change behaviour,^8^^,^S29^,^S33 whereas interactive decision-support tools, checklists, and patient-specific nudges have been shown to improve guideline adherence when information is tailored and clinically relevant.S66 Beyond technical design, uptake and durability depend on implementation planning, education, and organizational culture: sustained improvements typically require ongoing audit-and-feedback rather than one-off implementations.S33 The COORDINATE-Diabetes trial combined clinician education with audit-and-feedback to change prescribing practices.S67 Attitudes, perceived usefulness, and ease of use predict willingness to adopt clinical decision support systems more strongly than technical sophistication.S68 Finally, alert fatigue arises when clinicians are exposed to frequent, nonspecific notifications,S69 and the nuanced trade-offs embedded in clinical guidelines are often difficult to convey in brief prompts.

### Future Directions

Figure 1 proposes a structure for organizing efforts to improve the uptake and impact of nephrology guidelines across interdependent stages. First, guideline content should increasingly be developed in modular, machine-readable formats that support reuse across the platforms outlined above. Second, digital dissemination and education formats with demonstrated reach should be more deliberately aligned with guideline priorities and embedded within broader implementation plans, rather than deployed as standalone initiatives. Third, implementation strategies should be accompanied by predefined evaluation frameworks that incorporate routine monitoring of dissemination and uptake, ideally extending beyond usage metrics to assess effects on clinical processes, decision quality, and, where feasible, patient outcomes. In parallel, emerging AI-based approaches warrant cautious exploration, with emphasis on transparency, guideline fidelity, and human oversight, particularly in complex or preference-sensitive decisions.

**Figure 1:**
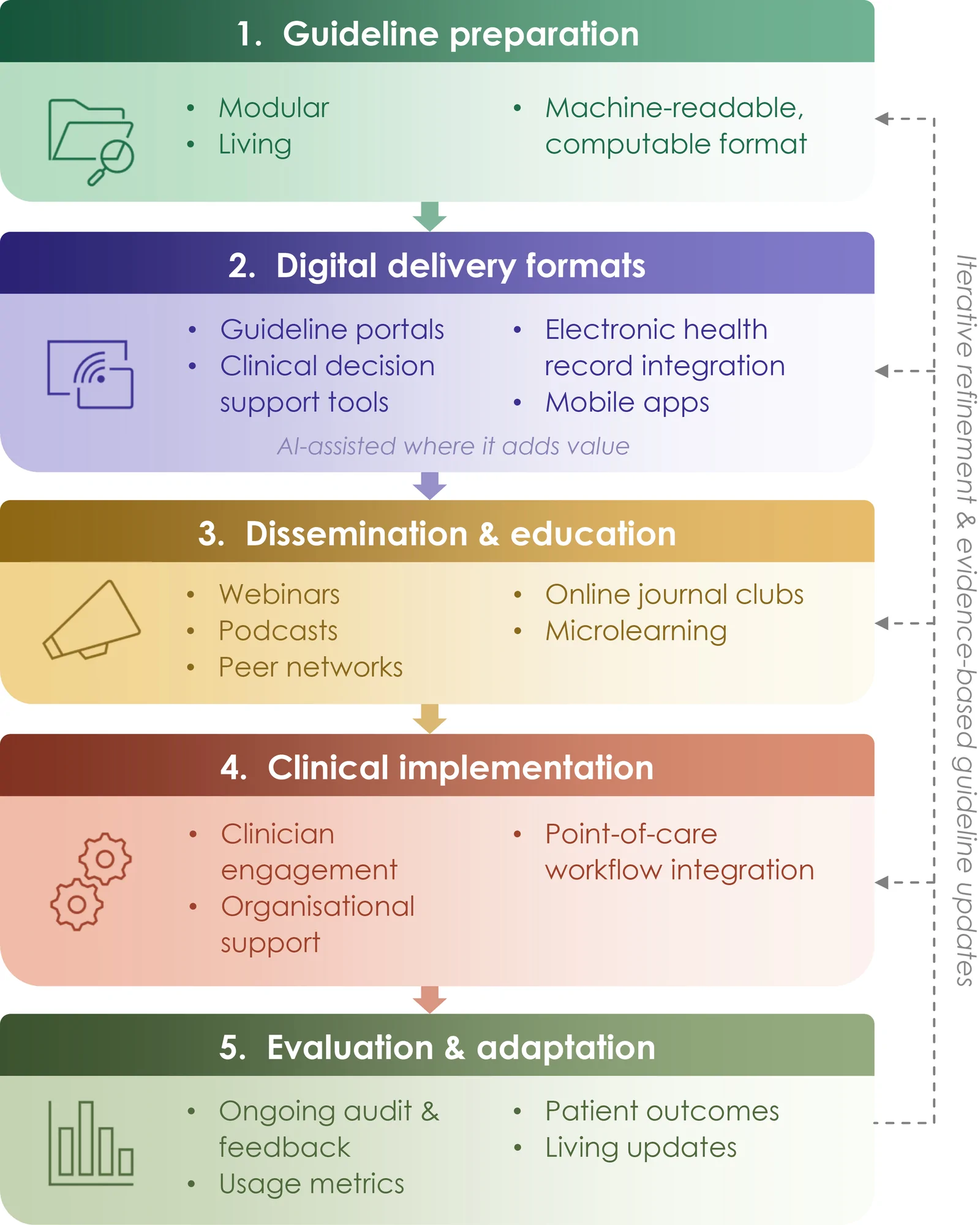
Proposed framework for digital guideline delivery.

## Conclusion

Digital approaches offer meaningful opportunities to improve the accessibility, usability, and reach of nephrology guidelines, but their impact depends on more than technical innovation alone. Digital decision-support tools that translate guideline recommendations to the point-of-care appear most effective when they are well embedded in clinical workflows and aligned with clinicians’ needs. Moving forward, digital guideline development should be complemented by coordinated dissemination, education, and implementation strategies, alongside systematic evaluation, to ensure that technological advances lead to meaningful improvements in clinical decision-making and patient care.

## Supplementary Material

Supplementary File (PDF) **Supplementary Methods. Supplementary References. Supplementary Text. Table S1.** Strengths and limitations of digital approaches to clinical practice guideline delivery.

## Footnotes

Supplementary File (PDF)

**Supplementary Methods.**

**Supplementary References.**

**Supplementary Text.**
**Table S1.** Strengths and limitations of digital approaches to clinical practice guideline delivery.
