# Supplementary material for: Rethinking Guideline Delivery in Nephrology: From Access to Action
Source: Kidney Int Rep. 2026 Sep 21;11(11):107089. doi: 10.1016/j.ekir.2026.107089 (PMC13677018; doi:10.1016/j.ekir.2026.107089)
Supplement: Supplementary file 1 [file mmc1.pdf]

Supplementary Material

Rethinking guideline delivery in nephrology: from access to action

Rob C.M. van Kruijsdijk, Tobias T. Pieters, Joel M. Topf, James O. Burton

---

Contents

- Supplementary Methods
- Supplementary Text S1: Detailed overview of digital approaches to clinical practice guideline delivery
- Supplementary Table S1: Strengths and limitations of digital approaches to clinical practice guideline delivery
- Supplementary References

Supplementary Methods

We conducted a literature search in PubMed on 30 October 2025 to identify existing papers on digital solutions aimed at improving the accessibility, usability, and implementation of clinical practice guidelines. The following search strategy was applied: ("Practice Guidelines as Topic"[Mesh] OR "clinical practice guideline"[tiab] OR "clinical guideline"[tiab] OR "practice guideline"[tiab] OR "evidence-based guideline"[tiab]) AND ("health information systems"[MeSH] OR "user-computer interface"[MeSH] OR "electronic health records"[MeSH] OR "digital"[Title/Abstract] OR "electronic"[Title/Abstract] OR "eHealth"[tiab] OR "clinical decision support"[tiab] OR "computerized decision support"[tiab] OR "electronic medical record*"[tiab] OR "machine-readable guideline*"[tiab] OR "e-learning"[tiab] OR "online learning"[tiab] OR "mobile applications"[MeSH] OR "mHealth"[tiab] OR "mobile health"[tiab] OR "artificial intelligence"[MeSH] OR "machine learning"[MeSH] OR "nudging"[tiab] OR "learning health system*"[tiab] OR "eHealth"[tiab] OR "decision aid*"[tiab] OR "risk calculator*"[tiab]) AND ("accessibility"[tiab] OR "usability"[tiab] OR "implementation"[tiab] OR "adoption"[tiab] OR "uptake"[tiab] OR "integration"[tiab]) NOT "Practice Guideline"[Publication Type].

The search was restricted to English-language articles published from 2015 onwards and retrieved 956 records. Records were eligible if they described or evaluated a digital approach to the presentation, dissemination, implementation, or point-of-care delivery of clinical practice guidelines. Records were divided among three authors (RvK, TP, JOB) for title and abstract screening. In total, 112 records were retrieved in full and selected for citation on the basis of relevance, study design, and the informativeness of the reported outcomes, with representative examples cited where several comparable studies addressed the same point, leaving 47 articles. A further 16 were identified through reference-list screening, targeted supplementary searches.

Furthermore, we conducted an inquiry into data on the accessibility and usage of clinical guidelines from nephrology organizations including KDIGO and UKKA. Data was requested on website traffic, user feedback, mobile app usage, media engagement, and survey results related to guideline implementation and use. Usage data from digital educational channels that discuss guideline-related topics, including the online journal club NephJC [https://www.nephjc.com/] and Freely Filtered podcasts, were also collected. Data were collected for a range of nephrology clinical practice guidelines published between 2014 and 2025 (*Table 1 and Supplementary Table S1*) 2025[S4–S19].

Reported usage counts reflect access events rather than unique individual users; counting conventions differ by platform. For KDIGO guidelines, usage metrics were generated using Google Analytics 4. Page views reflect the number of times a guideline page was viewed and file downloads the number of recorded download events for guideline PDFs and associated resources; both include repeat views/downloads by the same user and were not deduplicated by user or session. For guidelines published in BMC Nephrology (Springer Nature) and Peritoneal Dialysis International (SAGE) guidelines, repeated requests for the same article within a 30-second window are counted once, and known robot traffic is excluded; genuine repeat visits by the same reader are not deduplicated. For the 2014 hyponatraemia guideline (published in both the European Journal of Endocrinology and Nephrology Dialysis Transplantation, both hosted on Oxford Academic), the public 'Total Views' metric does not filter repeat views or exclude bot access. NephJC page views were obtained from Squarespace analytics and reflect the number of times a page was loaded; repeat views by the same visitor are counted separately and are not deduplicated by user or session.

Supplementary Text S1 | Detailed overview of digital approaches to clinical practice guideline delivery

Web-based guideline platforms

In nephrology, as in most medical specialties, guidelines are still primarily disseminated through static formats such as PDF documents or static webpages. While these allow open access, they offer limited functionality for navigation, updating, or usage monitoring, and thus limited support for real-time clinical decision-making. A study in anesthesiology demonstrated that migrating guideline content to structured web platforms can enhance accessibility and user tracking even without major technological investment[S1]. Within initiatives such as DECIDE and MAGIC, this approach has been further developed into modular and interactive web-based formats, using a multilayered electronic presentation in which clinicians can progressively expand recommendations to view their rationale and concise evidence summaries[S2]. User testing in six countries showed that participants generally found the multilayered format usable and visually appealing, but many struggled with core GRADE concepts such as distinguishing weak from strong recommendations. A subsequent cluster-randomized trial evaluated implementation of a schizophrenia guideline using either a traditional print/PDF format or an interactive MAGICapp-based format. Although no differences were observed in knowledge gain between formats, users of the interactive format reported better ease of use, faster learning, and greater improvements in decision-making[4]. In parallel, national guideline programs have begun to shift toward modular and updateable guidance. The National Institute for Health and Care Excellence has described a transition to natively digital, computable, and modular guideline content[5]. Such modular web-based architectures enable continuous updating and linkage with clinical data systems, thereby providing the technical foundation for truly living guideline models[S3].
Usage data from nephrology guideline organizations illustrate the relative contribution of different dissemination channels. For KDIGO guidelines, page views and PDF downloads via the organization's website substantially exceeded access via the journal website (*Table 1*), suggesting that the organizational website functions as the primary point of entry for guideline content for most users. User-reported preferences from a global KDIGO guideline survey (*n*=1,018) similarly indicate that concise, structured formats are most valued, with executive summaries and top 10 takeaways rated highest for practical utility in clinical practice[6].

Interactive and standalone decision support tools

Recent years have seen growing efforts to transform static guideline documents into interactive, user-oriented tools that support clinical reasoning and case-specific recommendations. These systems vary widely and include digital checklists and forms, visual encounter aids and web-based clinical decision support systems (CDSS).

A conceptual foundation for such tools lies in *knowledge-modeling* approaches, where guideline logic is encoded into interoperable digital structures that can combine multiple disease-specific recommendations into coherent, patient-tailored outputs[S20]. These frameworks provide a basis for scalable, transparent, and adaptable decision-support environments, forming the foundation for more advanced interactive systems.

Empirical studies across diverse settings demonstrate how interactivity and contextualization can enhance decision quality and adherence. In a randomized trial, an online HIV treatment platform doubled the accuracy of regimen selection compared with static guideline documents[S21]. In a Dutch intensive care unit, context-aware digital checklists increased compliance with best clinical practice items and slightly reduced ICU stay[S22]. Similarly, an electronic obstetric booking form embedding guideline logic improved compliance with gestational-age recommendations for elective caesarean

section, particularly among high-risk pregnancies[S23]. Examples from other clinical settings support the effectiveness of digital guideline tools. In primary care, encounter aids delivered through MAGICapp improved shared decision-making and patient understanding[S24], whereas in pediatrics, a guideline-based decision-support tool reduced unnecessary CT imaging[S25].

Beyond individual applications, a regional Spanish initiative developed a computational, model-driven system for type 2 diabetes that integrated cardiovascular and kidney-related recommendations, such as statin initiation in patients with chronic kidney disease (CKD). System-generated advice showed high concordance with clinician decisions[S26], illustrating feasibility for chronic-disease management at scale, although the evaluation was limited by variability in electronic health record (EHR) data quality and the absence of direct assessment of clinical outcomes.

Across these examples, improvements were context dependent and often modest. Many tools primarily affected process measures rather than hard outcomes, and several interventions achieved significance only for specific subgroups or metrics[S23, S25, S26]. Time constraints, data completeness, and limited system integration restricted broader use of encounter aids and model-driven tools[S22, S24, S26], while implementation support and clinician education could enable effectiveness[S27]. These findings highlight that the success of interactive CDSS depends not only on the underlying algorithm but also on workflow alignment, user engagement, education and implementation support.

Electronic Health Record-integrated decision support

Integrating clinical guidelines within EHRs represents a crucial step toward bringing evidence-based recommendations directly to the point of care. In practice, EHR-integrated guideline support has been implemented through a wide range of tools, including point-of-care decision support, structured order sets and documentation workflows, and system-level feedback such as dashboards.

Several real-world evaluations illustrate that decision support that is well integrated into the clinical workflow can lead to measurable changes in clinician behavior and patient outcomes. In primary care, a pragmatic cluster-randomized evaluation in China tested a guideline-based decision-support system for hypertension that provided patient-specific medication advice in real time which increased adherence to recommended therapy by 15.2% and resulted in a modest blood pressure reduction[7]. A telemetry-ordering intervention that introduced a context-aware alert requiring justification for non-indicated monitoring cut unnecessary telemetry[S28].

However, technical integration alone is not sufficient. For example, the NUDGE-CKD trial, using digital letters to encourage patients with CKD and their general practitioners to start recommended therapies, showed no effect on prescribing rates[8]. The intervention reached tens of thousands of individuals but was neither embedded in the EHR nor personalized to clinical encounters, demonstrating that scale alone cannot compensate for lack of integration, timing, or context. Similar limitations were observed in a large multicenter cluster-randomized study that implemented a non-interruptive EHR alert and order set to prompt complete evaluation of high-risk microscopic hematuria but failed to improve adherence[S29]. Although the alert fired correctly, it lacked personalization, appeared outside the diagnostic sequence, and required coordination across multiple departments, which limited its influence. These examples highlight that alerts must be specific, actionable, and timed to the clinician's cognitive workflow rather than existing as isolated informational reminders. A more deeply embedded approach involves coupling guideline logic directly to order sets and structured documentation, which can standardize complex care processes and improve adherence. However, effectiveness still depends on workflow alignment, usability, ongoing updates, and clinician ownership. For example, a multicenter trauma initiative linking venous-thromboembolism prophylaxis guidelines to an admission order set achieved both higher adherence and lower event rates[S30], and integration of a colonoscopy-surveillance module into routine reporting improved adherence to follow-up recommendations[S31].

Several tools combine guideline logic with continuous feedback and benchmarking to reinforce adherence over time. Near real-time performance dashboards embedded in anesthesia information systems, supplemented with individual and peer-comparison feedback, achieved sustained improvements in lung-protective ventilation strategies[S32]. Team-based audit-and-feedback dashboards using continuously updated EHR data similarly improved inpatient quality metrics, although effects attenuated once active feedback ceased[S33]. In primary care, EHR-linked dashboards supporting audit, benchmarking and facilitated quality-improvement planning modestly improved guideline-recommended testing for CKD, diabetes and cardiovascular disease[S27]. Across settings, systematic evaluations indicate that dashboards are most effective when combined with ongoing feedback cycles, benchmarking, and organizational or educational support rather than standalone visualization tools[S34, S35].

Mobile applications

Since smartphones are now ubiquitous among clinicians and patients, mobile apps offer a promising platform for delivering guideline-derived algorithms, risk scores, and CDSS during clinical care. Their portability makes them particularly useful in time-pressured environments, such as the emergency department, or environments where there is limited access to computers or certain software, such as low-resource contexts. Mobile apps can also engage patients directly - either by providing clinicians with structured information using questionnaires before a clinical consult or providing them with guideline-based educational content.

Mobile applications have been used to operationalize guidelines for a variety of clinical contexts with studies showing encouraging effects on application use and effect on guideline adherence. For example, a study evaluating a mobile clinical decision aid for pediatric sepsis demonstrated rapid dissemination and in-depth user engagement[S36], whereas another CDSS for pediatric asthma care showed reduced odds of hospital admission and greater referral to smoking cessation with increased use of the app[S37]. In low-resource and rural settings mobile apps have been suggested as pragmatic tools to extend access to guideline-based recommendations and standardize guideline-based treatment where specialist support or computer infrastructure is limited[S38–S40]. These findings are consistent with user-reported preferences: in the KDIGO guideline survey 80.5% indicated that a mobile application with easy access to guidelines would be a valued additional resource[6].

In parallel with these tools for clinicians, an expanding number of patient-oriented self-management apps have been developed. These applications typically target behavioral domains such as diet, medication adherence, symptom tracking, and general self-management. Randomized and prospective studies suggest that such apps are feasible and may improve self-management behaviors, with potential benefits for blood-pressure control and medication adherence in patients with CKD, albeit with heterogeneity in study quality and duration[S41–S43].

Although apps are increasingly used there are limitations. Overall, studies on the use of mobile apps mainly report the initial uptake and effect on guideline adherence, whilst the studies that report an effect of clinical outcomes are mainly limited to before/after studies and report modest effects. Some articles note that initial uptake is often high, but long-term engagement declines[S44–S46]. Clinicians report that apps perceived as outdated, slow, or poorly aligned with their routines quickly fall out of use[S37, S38]. Additional barriers, such as inconsistent device access, digital literacy, and connectivity issues in low-resource settings, further limit consistent use over time[S40].

At present, there are no peer-reviewed articles examining the uptake, implementation, or clinical effect of the major nephrology guideline-based mobile applications. The National Kidney Foundation (NKF) has developed the KDOQI App, which provides clinicians with mobile access to KDOQI clinical practice guidelines and accompanying commentaries, and has also released a separate decision-support tool—the myVascularAccess app—to support vascular access planning for patients receiving

hemodialysis. Similarly, the KDIGO Mobile App, first launched in the early 2010s, is no longer available but offered a comparable function. More recently, the European Rare Kidney Disease Reference Network (ERKNet) has introduced the ERKNet/LeiLa Guideline App, which encompasses rare kidney disease guidelines, pediatric nephrology recommendations, and diagnostic decision pathways.

Artificial intelligence

Artificial intelligence (AI) holds significant promise for enhancing the adoption and dissemination of clinical guidelines, helping clinicians access guideline-based information in various formats and aiding patients in understanding these guidelines in layman's terms. AI-driven models can support clinicians in multiple ways, with varying levels of sophistication and specificity, from extraction of relevant guidelines sections to AI-driven decision support. Moreover, numerous underlying models can be applied in these settings, each with their own advantages and limitations. Given the rapid advancements in AI technology, it is unlikely that peer-reviewed research can keep pace with the latest developments, nor that the accuracy of AI models will be thoroughly evaluated for every existing guideline. Despite these concerns, the widespread availability of AI-driven models has led to their increasing use in clinical practice, with a recent American Medical Association survey indicating that 66% of physicians report utilizing AI tools regularly in their practice[S47].

A wide range of AI technologies supporting guideline-directed care is represented across the literature. Several studies compare general-purpose large language models (LLMs) such as ChatGPT, DeepSeek, Gemini, and Claude in tasks like staging cancers, recommending treatment plans, or generating patient management advice[S48–S53]. Others deploy retrieval-augmented generation (RAG) systems that integrate guideline text directly into the model context, enabling more transparent and verifiable outputs[S57, S58]. One study showed that large language models can automatically extract key eligibility criteria and recommended actions from preventive guidelines, enabling the generation of structured decision support logic[S60].

When compared with clinician-based consensus or guidelines, AI-driven models show variable performance, with generally moderate-to-high guideline adherence across oncology use cases[S53–S56] and more heterogeneous results in other clinical domains such as acute cholecystitis[S49], musculoskeletal pain[S49], and imaging referral decisions[S51]. A study evaluating common hypertension management scenarios showed that, while LMM generated guideline-concordant recommendations in a substantial proportion of cases, its accuracy and safety remained inferior to expert decisions, underscoring the need for human-in-the-loop oversight[S61]. Although none of these studies directly assessed patient outcomes, several report potential downstream benefits through improved consistency, accessibility, and safety of guideline-based care, particularly for retrieval-augmented models, which reduced hallucinations and improved accuracy[S57–S59].

Despite promising results, important limitations remain. Inaccurate or misleading recommendations were reported across models, particularly in complex staging or guideline interpretation. Most studies relied on simulated vignettes rather than real-world clinical use, and none compared these systems with established, rule-based decision support tools. Rapid guideline updates further challenge reliability when models are not continuously updated. In addition, heterogeneity in study methods limits comparability, and regulatory, medicolegal, and workflow implications have largely not been addressed. As a result, stronger clinical evidence and standardized evaluation are needed before routine implementation can be recommended.

Digital dissemination and education tools

Guideline organizations have traditionally relied on dissemination-focused strategies that are feasible and scalable, yet these approaches are rarely accompanied by formal evaluation of downstream clinical impact[3]. Beyond decision-support technologies, a growing ecosystem of digital education and dissemination formats contributes to the reach of clinical practice guidelines. Webinars, online journal clubs, podcasts, and microlearning formats enable low-threshold, on-demand engagement with guideline-related content and facilitate discussion of recommendations in clinical context. Within nephrology, social-media–enabled education initiatives such as NephJC exemplify how online journal clubs can achieve sustained international participation and broad dissemination, extending critical appraisal and discussion well beyond local institutions[9, S62, S63]. Reports from other specialties similarly shows that social media-based journal clubs substantially increase engagement metrics, compared with traditional dissemination alone[S70, S71].

Podcasts represent another widely used dissemination format, valued by clinicians for their accessibility, flexibility, and ability to contextualize complex topics, with large download volumes reported across medical specialties[S64]. Microlearning approaches, ranging from brief app-based case or quiz modules to bite-sized formats such as short podcasts, microblogs, and visual summaries, are associated with high learner engagement and short-term gains in knowledge and confidence, although the effects on clinician behavior rarely evaluated.[S65].
Usage metrics summarized in *Table 1* illustrate that these digital formats can achieve substantial reach, supporting their role as complementary dissemination tools that may contribute to broader awareness and in-depth understanding of guideline recommendations. User perspectives from the KDIGO guideline survey indicate substantial demand for digital learning formats, with online training modules (53%) and educational webinars (42%) among the most frequently requested additional resources for guideline implementation[6].

Barriers and Facilitators

New digital technologies hold substantial promise for improving the dissemination and implementation of clinical guidelines, yet their integration into routine medical practice remains slow. Modular web-based platforms improve accessibility and user satisfaction and offer the advantage of continuous updating, supporting living guideline models[S3]. Evidence across studies evaluating digital approaches to bring guidelines to the point-of-care indicate these tools are most effective when they are delivered at the moment of decision, provide concise and patient-specific recommendations, and link directly to executable actions such as orders or documentation templates[7, S28, S30–S32]. Tools that operate outside the clinical workflow or rely on passive reminders rarely change behaviour[8, S29, S33].

Interactivity and contextualization are equally important: interactive decision-support tools, checklists, and patient-specific nudges have been shown to improve guideline adherence when information is tailored and clinically relevant, as demonstrated in the NOTIFY-1 trial using individualized coronary artery calcium imaging with guideline-based recommendations[S66]. Conversely, effects are blunted when information is poorly timed, insufficiently specific, or misaligned with workflow. This is illustrated by large trials of CDSS for high-risk hematuria[S29] and digital nudges for CKD management in NUDGE-CKD[8], neither of which significantly altered clinicians practice.

Beyond technical design, uptake and durability depend on implementation planning, education, and organizational culture. Change-management frameworks emphasize the importance of creating urgency, clear communication, and embedding new practices as the norm. Consistent with these principles, sustained improvements typically require ongoing audit-and-feedback rather than one-off implementations, as illustrated by EHR-based interventions in which gains diminished once feedback was withdrawn[S33]. Consistent with these principles, the COORDINATE-Diabetes trial showed that a

multifaceted intervention combining clinician education with audit-and-feedback successfully changed prescribing practices[S67]. Similarly, a recent Thai survey found that attitudes, perceived usefulness, and ease of use were the strongest predictors of willingness to adopt preventive-care CDSS[S68], underscoring that trust and clinical relevance are as critical as technical sophistication.

Finally, increasing volumes of alerts and recommendations may themselves become a barrier. Alert fatigue arises when clinicians are exposed to frequent, nonspecific notifications[S69], and the nuanced trade-offs embedded in clinical guidelines are often difficult to convey in brief prompts. These concerns extend to emerging AI-based approaches, including large language models that generate tailored recommendations from guideline content. Whether such systems can reliably preserve guideline fidelity and nuance in real-world use remains an open question.

Supplementary Table S1 | Strengths and limitations of digital approaches to clinical practice guideline delivery

DIGITAL APPROACH	STRENGTHS	LIMITATIONS	KEY REFERENCES
**STATIC WEB-BASED GUIDELINES (PDFS, BASIC WEBPAGES)**	Broad accessibility; low development cost; familiar format	Limited navigability and updating; no contextualization or workflow integration	1, 2, S1
**MODULAR / INTERACTIVE WEB-BASED PLATFORMS**	Improved usability and navigation; supports living guidelines; enables usage analytics	Requires active access by users; limited point-of-care integration	4, 5, S2, S3
**STANDALONE INTERACTIVE DECISION-SUPPORT TOOLS**	Translates guidelines into patient-specific recommendations; supports decision-making and adherence	Variable impact on outcomes; time burden; effectiveness depends on workflow fit	S20–S26
**EHR-INTEGRATED DECISION SUPPORT (ALERTS, ORDER SETS)**	Point-of-care delivery; actionable recommendations; demonstrated effects on clinician behavior	Alert fatigue; high technical complexity; poor design limits effectiveness	7, 8, S28–S31
**AUDIT-AND-FEEDBACK DASHBOARDS**	Reinforces adherence over time; enables benchmarking and quality improvement	Limited effect without sustained feedback; dependent on data quality and organizational support	S27, S32–S35
**MOBILE APPLICATIONS (CLINICIAN-FOCUSED)**	Portable and accessible; useful in time-pressured or low-resource settings	Declining long-term engagement; limited integration; risk of outdated content	S36–S40, S44–S46
**MOBILE APPLICATIONS (PATIENT-FOCUSED)**	Supports education and self-management; may improve adherence behaviors	Heterogeneous evidence; modest clinical effects; digital literacy barriers	S41–S43
**AI-ASSISTED GUIDELINE TOOLS**	Flexible access to guideline content; potential for personalized recommendations at scale	Variable accuracy; limited validation; safety, regulatory, and governance concerns	S47–S61
**DIGITAL DISSEMINATION AND EDUCATION TOOLS**	High reach; low threshold for engagement; contextualizes guideline content	Limited impact on clinician behavior or outcomes when used alone	9, S62–S65, S70, S71

Supplementary References

S1. Bachmann KF, Vetter C, Wenzel L*, et al.* Implementation and Evaluation of a Web-Based Distribution System For Anesthesia Department Guidelines and Standard Operating Procedures: Qualitative Study and Content Analysis. *J Med Internet Res* 2019; 21: e14482.

S2. Kristiansen A, Brandt L, Alonso-Coello P*, et al.* Development of a novel, multilayered presentation format for clinical practice guidelines. *Chest* 2015; 147: 754–763.

S3. Pielenz C, Schneider M, Salveridou-Hof E*, et al.* From conventional to living guidelines - faster updates for better informed guidance? A scoping review. *Z Evid Fortbild Qual Gesundhwes* 2022; 174: 20–31.

S4. Doulton T, Adam M, Durman K*, et al.* Management of blood pressure in adults, children and young people on dialysis: UK kidney association clinical practice guideline. *BMC Nephrol* 2025; 26: 532.

S5. Kidney Disease: Improving Global Outcomes Ig AN, Ig AVWG, Rovin BH*, et al.* KDIGO 2025 Clinical Practice Guideline for the Management of Immunoglobulin A Nephropathy (IgAN) and Immunoglobulin A Vasculitis (IgAV). *Kidney Int* 2025; 108: S1–S71.

S6. Aitken E, Anijeet H, Ashby D*, et al.* UK Kidney Association Clinical Practice Guideline on vascular access for haemodialysis. *BMC Nephrol* 2025; 26: 461.

S7. Bhandari S, Spencer S, Oliveira B*, et al.* UK kidney association clinical practice guideline: update of anaemia of chronic kidney disease. *BMC Nephrol* 2025; 26: 193.

S8. Kidney Disease: Improving Global Outcomes AWG. KDIGO 2025 Clinical Practice Guideline for the Evaluation, Management, and Treatment of Autosomal Dominant Polycystic Kidney Disease (ADPKD). *Kidney Int* 2025; 107: S1–S239.

S9. Zac-Varghese S, Mark P, Bain S*, et al.* Clinical practice guideline for the management of lipids in adults with diabetic kidney disease: abbreviated summary of the Joint Association of British Clinical Diabetologists and UK Kidney Association (ABCD-UKKA) Guideline 2024. *BMC Nephrol* 2024; 25: 216.

S10. Kidney Disease: Improving Global Outcomes CKDWG. KDIGO 2024 Clinical Practice Guideline for the Evaluation and Management of Chronic Kidney Disease. *Kidney Int* 2024; 105: S117–S314.

S11. Kidney Disease: Improving Global Outcomes Lupus Nephritis Work G. KDIGO 2024 Clinical Practice Guideline for the management of LUPUS NEPHRITIS. *Kidney Int* 2024; 105: S1–S69.

S12. Li PK, Chow KM, Cho Y*, et al.* ISPD peritonitis guideline recommendations: 2022 update on prevention and treatment. *Perit Dial Int* 2022; 42: 110–153.

S13. Kidney Disease: Improving Global Outcomes Glomerular Diseases Work G. KDIGO 2021 Clinical Practice Guideline for the Management of Glomerular Diseases. *Kidney Int* 2021; 100: S1–S276.

S14. Kidney Disease: Improving Global Outcomes Blood Pressure Work G. KDIGO 2021 Clinical Practice Guideline for the Management of Blood Pressure in Chronic Kidney Disease. *Kidney Int* 2021; 99: S1–S87.

S15. Kidney Disease: Improving Global Outcomes Diabetes Work G. KDIGO 2020 Clinical Practice Guideline for Diabetes Management in Chronic Kidney Disease. *Kidney Int* 2020; 98: S1–S115.

S16. Nourse P, Cullis B, Finkelstein F*, et al.* ISPD guidelines for peritoneal dialysis in acute kidney injury: 2020 Update (paediatrics). *Perit Dial Int* 2021; 41: 139–157.

S17. Whelton PK, Carey RM, Aronow WS*, et al.* 2017 ACC/AHA/AAPA/ABC/ACPM/AGS/APhA/ASH/ASPC/NMA/PCNA Guideline for the Prevention, Detection, Evaluation, and Management of High Blood Pressure in Adults: A Report of the American College of Cardiology/American Heart Association Task Force on Clinical Practice Guidelines. *Hypertension* 2018; 71: e13–e115.

S18. Spasovski G, Vanholder R, Allolio B*, et al.* Clinical practice guideline on diagnosis and treatment of hyponatraemia. *Eur J Endocrinol* 2014; 170: G1–47.

S19. Spasovski G, Vanholder R, Allolio B*, et al.* Clinical practice guideline on diagnosis and treatment of hyponatraemia. *Nephrol Dial Transplant* 2014; 29 Suppl 2: i1–i39.

S20. Abidi S. A Knowledge-Modeling Approach to Integrate Multiple Clinical Practice Guidelines to Provide Evidence-Based Clinical Decision Support for Managing Comorbid Conditions. *J Med Syst* 2017; 41: 193.

S21. Ramirez JA, Maddali MV, Nematollahi S*, et al.* New Strategies in Clinical Guideline Delivery: Randomized Trial of Online, Interactive Decision Support Versus Guidelines for Human Immunodeficiency Virus Treatment Selection by Trainees. *Clin Infect Dis* 2021; 72: 1608–1614.

S22. De Bie AJR, Mestrom E, Compagner W*, et al.* Intelligent checklists improve checklist compliance in the intensive care unit: a prospective before-and-after mixed-method study. *Br J Anaesth* 2021; 126: 404–414.

S23. Vitali J, Janssens S, Ravi N*, et al.* Did the introduction of an electronic booking form for elective caesarean section improve compliance with guidelines for gestational age at delivery? *Int J Qual Health Care* 2025; 37.

S24. Van Bostraeten P, Aertgeerts B, Bekkering G*, et al.* Digital encounter decision aids linked to clinical practice guidelines: results from user testing SHARE-IT decision aids in primary care. *BMC Med Inform Decis Mak* 2023; 23: 97.

S25. Ballard DW, Kuppermann N, Vinson DR*, et al.* Implementation of a Clinical Decision Support System for Children With Minor Blunt Head Trauma Who Are at Nonnegligible Risk for Traumatic Brain Injuries. *Ann Emerg Med* 2019; 73: 440–451.

S26. Parra-Calderon CL, Roman-Villaran E, Alvarez-Romero C*, et al.* A prospective observational concordance study to evaluate computational model-driven clinical practice guidelines for Type 2 diabetes mellitus. *Int J Med Inform* 2023; 178: 105208.

S27. Jones JL, Simons K, Manski-Nankervis JA*, et al.* Chronic disease IMPACT (chronic disease early detection and improved management in primary care project): An Australian stepped wedge cluster randomised trial. *Digit Health* 2023; 9: 20552076231194948.

S28. Bergstedt A, Hilliard B, Alabsi S*, et al.* Evaluation of a Clinical Decision Support Tool to Guide Adoption of the American Heart Association Telemetry Monitoring Practice Standards. *J Am Heart Assoc* 2024; 13: e031523.

S29. Matulewicz RS, Tsuruo S, King WC*, et al.* Efficacy of a Clinical Decision Support Tool to Promote Guideline-Concordant Evaluations in Patients With High-Risk Microscopic Hematuria: A Cluster Randomized Quality Improvement Project. *J Urol* 2025; 213: 558–567.

S30. Lee JS, Johnson E, Schmoekel NH*, et al.* Impact of implementing a venous thromboembolism guideline and electronic health records order set on venous thromboembolism rates in trauma patients: A multicenter study. *J Trauma Acute Care Surg* 2025; 99: 212–218.

S31. Magrath M, Yang E, Ahn C*, et al.* Impact of a Clinical Decision Support System on Guideline Adherence of Surveillance Recommendations for Colonoscopy After Polypectomy. *J Natl Compr Canc Netw* 2018; 16: 1321–1328.

S32. Parks DA, Short RT, McArdle PJ*, et al.* Improving Adherence to Intraoperative Lung-Protective Ventilation Strategies Using Near Real-Time Feedback and Individualized Electronic Reporting. *Anesth Analg* 2021; 132: 1438–1449.

S33. Patel S, Rajkomar A, Harrison JD*, et al.* Next-generation audit and feedback for inpatient quality improvement using electronic health record data: a cluster randomised controlled trial. *BMJ Qual Saf* 2018; 27: 691–699.

S34. Meier FL, Jager L, Senn O*, et al.* Effectiveness of interactive dashboards as audit and feedback tools in primary care: A systematic review. *PLoS One* 2025; 20: e0327350.

S35. Xie CX, Chen Q, Hincapie CA*, et al.* Effectiveness of clinical dashboards as audit and feedback or clinical decision support tools on medication use and test ordering: a systematic review of randomized controlled trials. *J Am Med Inform Assoc* 2022; 29: 1773–1785.

S36. McCulloh RJ, Fouquet SD, Herigon J*, et al.* Development and implementation of a mobile device-based pediatric electronic decision support tool as part of a national practice standardization project. *J Am Med Inform Assoc* 2018; 25: 1175–1182.

S37. Kerns E, McCulloh R, Fouquet S*, et al.* Utilization and effects of mobile electronic clinical decision support on pediatric asthma care quality in the emergency department and inpatient setting. *JAMIA Open* 2021; 4: ooab019.

S38. Baykemagn ND, Nigatu AM, Fikadie B*, et al.* Acceptance of mobile application-based clinical guidelines among health professionals in Northwestern Ethiopia: A mixed-methods study. *Digit Health* 2024; 10: 20552076241261930.

S39. Orchard J, Li J, Freedman B*, et al.* Atrial Fibrillation Screen, Management, and Guideline-Recommended Therapy in the Rural Primary Care Setting: A Cross-Sectional Study and Cost-Effectiveness Analysis of eHealth Tools to Support All Stages of Screening. *J Am Heart Assoc* 2020; 9: e017080.

S40. Usmanova G, Lalchandani K, Srivastava A*, et al.* The role of digital clinical decision support tool in improving quality of intrapartum and postpartum care: experiences from two states of India. *BMC Pregnancy Childbirth* 2021; 21: 278.

S41. Li R, Liang N, Bu F*, et al.* The Effectiveness of Self-Management of Hypertension in Adults Using Mobile Health: Systematic Review and Meta-Analysis. *JMIR Mhealth Uhealth* 2020; 8: e17776.

S42. Ghozali MT, Satibi S, Forthwengel G. The impact of mobile health applications on the outcomes of patients with chronic kidney disease: a systematic review and meta-analysis. *J Med Life* 2023; 16: 1299–1309.

S43. Paneerselvam GS, Lua PL, Chooi WH*, et al.* Effectiveness of Mobile Apps in Improving Medication Adherence Among Chronic Kidney Disease Patients: Systematic Review. *J Med Internet Res* 2025; 27: e53144.

S44. Matthews S, Atkins B, Walton N*, et al.* Development and Use of a Cardiac Clinical Guideline Mobile App in Australia: Acceptability and Multi-Methods Study. *JMIR Form Res* 2022; 6: e35599.

S45. Gupta RK, McEvoy MD. Initial Experience of the American Society of Regional Anesthesia and Pain Medicine Coags Regional Smartphone Application: A Novel Report of Global Distribution and Clinical Usage of an Electronic Decision Support Tool to Enhance Guideline Use. *Reg Anesth Pain Med* 2016; 41: 334–338.

S46. Meidani Z, Atoof F, Mobarak Z, *et al.* Development of clinical-guideline-based mobile application and its effect on head CT scan utilization in neurology and neurosurgery departments. *BMC Med Inform Decis Mak* 2022; 22: 106.

S47. American Medical Association. AMA augmented intelligence research: Physician sentiments around the use of AI in health care—motivations, opportunities, risks, and use cases. Shifts from 2023 to 2024.

S48. Dong C, Qiu X, Deng J, *et al.* Comparative evaluation of large language models in delivering guideline-compliant recommendations for topical NSAID use in musculoskeletal pain: a multidimensional analysis. *Clin Rheumatol* 2025; 44: 4703–4710.

S49. Goglia M, Cicolani A, Carrano FM, *et al.* Using Large Language Models in the Diagnosis of Acute Cholecystitis: Assessing Accuracy and Guidelines Compliance. *Am Surg* 2025; 91: 967–977.

S50. Gunesli I, Aksun S, Fathelbab J, *et al.* Comparative evaluation of ChatGPT-4, ChatGPT-3.5 and Google Gemini on PCOS assessment and management based on recommendations from the 2023 guideline. *Endocrine* 2025; 88: 315–322.

S51. Potocnik J, Thomas E, Kearney D, *et al.* Can ChatGPT and Gemini justify brain CT referrals? A comparative study with human experts and a custom prediction model. *Eur Radiol Exp* 2025; 9: 24.

S52. Safran E, Yildirim S. A cross-sectional study on ChatGPT's alignment with clinical practice guidelines in musculoskeletal rehabilitation. *BMC Musculoskelet Disord* 2025; 26: 411.

S53. Vural Camalan B, Doluoglu S, Taraf NH, *et al.* ChatGPT versus DeepSeek in head and neck cancer staging and treatment planning: guideline-based study. *Eur Arch Otorhinolaryngol* 2025; 282: 4815–4824.

S54. Kaiser KN, Hughes AJ, Yang AD, *et al.* Use of large language models as clinical decision support tools for management pancreatic adenocarcinoma using National Comprehensive Cancer Network guidelines. *Surgery* 2025; 182: 109267.

S55. Piazza D, Martorana F, Curaba A, *et al.* The Consistency and Quality of ChatGPT Responses Compared to Clinical Guidelines for Ovarian Cancer: A Delphi Approach. *Curr Oncol* 2024; 31: 2796–2804.

S56. Pereyra L, Schlottmann F, Steinberg L, *et al.* Colorectal Cancer Prevention: Is Chat Generative Pretrained Transformer (Chat GPT) ready to Assist Physicians in Determining Appropriate Screening and Surveillance Recommendations? *J Clin Gastroenterol* 2024; 58: 1022–1027.

S57. Giuffre M, Pugliese N, Kresevic S*, et al.* From Guidelines to Real-Time Conversation: Expert-Validated Retrieval-Augmented and Fine-Tuned GPT-4 for Hepatitis C Management. *Liver Int* 2025; 45: e70349.

S58. Goh SSN, Mariappan R, Soo Woon Tan G*, et al.* Augmenting Large Language Models With National Comprehensive Cancer Network Guidelines for Improved and Standardized Adjuvant Therapy Recommendations in Postoperative Breast Cancer Cases. *JCO Clin Cancer Inform* 2025; 9: e2400243.

S59. Zhao Z, An B, Zhang T*, et al.* Integrating clinical guidelines with large language models for improved sepsis mortality prediction. *Health Informatics J* 2025; 31: 14604582251387649.

S60. Luo X, Tahabi FM, Marc T*, et al.* Zero-shot learning to extract assessment criteria and medical services from the preventive healthcare guidelines using large language models. *J Am Med Inform Assoc* 2024; 31: 1743–1753.

S61. Zand J, Miao J, Hommos MS*, et al.* Performance of Large Language Models in Analyzing Common Hypertension Scenarios. *Hypertension* 2026; 83: 225–234.

S62. Shankar M, Sparks MA. The evolution of social media in nephrology education: A mini-review. *Front Nephrol* 2023; 3: 1123969.

S63. Topf JM, Sparks MA, Phelan PJ*, et al.* The Evolution of the Journal Club: From Osler to Twitter. *Am J Kidney Dis* 2017; 69: 827–836.

S64. Newman J, Liew A, Bowles J*, et al.* Podcasts for the Delivery of Medical Education and Remote Learning. *J Med Internet Res* 2021; 23: e29168.

S65. De Gagne JC, Park HK, Hall K*, et al.* Microlearning in Health Professions Education: Scoping Review. *JMIR Med Educ* 2019; 5: e13997.

S66. Sandhu AT, Rodriguez F, Ngo S*, et al.* Incidental Coronary Artery Calcium: Opportunistic Screening of Previous Nongated Chest Computed Tomography Scans to Improve Statin Rates (NOTIFY-1 Project). *Circulation* 2023; 147: 703–714.

S67. Pagidipati NJ, Nelson AJ, Kaltenbach LA*, et al.* Coordinated Care to Optimize Cardiovascular Preventive Therapies in Type 2 Diabetes: A Randomized Clinical Trial. *JAMA* 2023; 329: 1261–1270.

S68. Sitasuwan T, Suriyaphol P, Lawpoolsri S*, et al.* Factors Influencing Health Workers' Acceptance of Guideline-Based Clinical Decision Support Systems for Preventive Services in Thailand: Questionnaire-Based Study. *JMIR Hum Factors* 2025; 12: e57314.

S69. Olakotan O, Mohd Yusof M, Ezat Wan Puteh S. A Systematic Review on CDSS Alert Appropriateness. *Stud Health Technol Inform* 2020; 270: 906–910.

S70. Sarwal A, Desai M, Juneja P*, et al.* Twitter Journal Club Impact on Engagement Metrics of the Neurocritical Care Journal. *Neurocrit Care* 2022; 37: 129–139.

S71. Schwartz IS, McCarty T, Woc-Colburn LE*, et al.* Educational Impact of #IDJClub, a Twitter-Based Infectious Diseases Journal Club. *Clin Infect Dis* 2022; 74: S244–S250.
